# The Inflammatory Actions of Coagulant and Fibrinolytic Proteases in Disease

**DOI:** 10.1155/2015/437695

**Published:** 2015-03-24

**Authors:** Michael Schuliga

**Affiliations:** Lung Health Research Centre, Department of Pharmacology and Therapeutics, University of Melbourne, Parkville, VIC 3010, Australia

## Abstract

Aside from their role in hemostasis, coagulant and fibrinolytic proteases are important mediators of inflammation in diseases such as asthma, atherosclerosis, rheumatoid arthritis, and cancer. The blood circulating zymogens of these proteases enter damaged tissue as a consequence of vascular leak or rupture to become activated and contribute to extravascular coagulation or fibrinolysis. The coagulants, factor Xa (FXa), factor VIIa (FVIIa), tissue factor, and thrombin, also evoke cell-mediated actions on structural cells (e.g., fibroblasts and smooth muscle cells) or inflammatory cells (e.g., macrophages) via the proteolytic activation of protease-activated receptors (PARs). Plasmin, the principle enzymatic mediator of fibrinolysis, also forms toll-like receptor-4 (TLR-4) activating fibrin degradation products (FDPs) and can release latent-matrix bound growth factors such as transforming growth factor-*β* (TGF-*β*). Furthermore, the proteases that convert plasminogen into plasmin (e.g., urokinase plasminogen activator) evoke plasmin-independent proinflammatory actions involving coreceptor activation. Selectively targeting the receptor-mediated actions of hemostatic proteases is a strategy that may be used to treat inflammatory disease without the bleeding complications of conventional anticoagulant therapies. The mechanisms by which proteases of the coagulant and fibrinolytic systems contribute to extravascular inflammation in disease will be considered in this review.

## 1. Introduction

**Figure 1:**
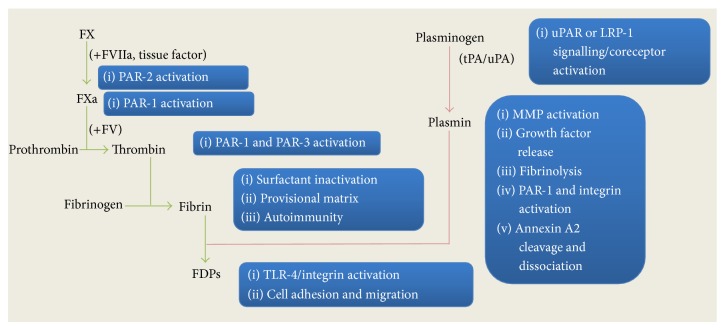
The proinflammatory and proremodelling actions of coagulant and fibrinolytic proteases. In tissue injury and disease, the proteases FXa, FVIIa, thrombin, plasmin, uPA, and tPA not only participate in extravascular coagulation (green pathway) or fibrinolysis (red pathway), but also mediate inflammation and tissue remodelling. The cell-mediated actions of the individual proteases can overlap (i.e., PAR-1 activation by FXa, thrombin, and plasmin) and/or be interconnected (i.e., the generation of FDPs by both coagulation and fibrinolytic pathways).

As part of hemostasis, the coagulation and fibrinolytic pathways regulate vascular repair by forming and degrading fibrin, respectively [1]. In disease, increased vascular permeability allows blood-circulating hemostatic factors such as factor X (FX) and plasminogen to enter damaged tissue to become activated and participate in coagulation or fibrinolysis. Fibrin deposition within damaged tissues is a common pathological feature and evidence of extravascular coagulation in inflammatory diseases including many respiratory and vascular diseases [2–5], rheumatoid arthritis [6], and cancer [7]. Aside from roles in fibrin homeostasis, several of the coagulant and fibrinolytic proteases exert potent proinflammatory and proremodelling actions in disease. These actions may be independent of fibrin formation, such as the activation of protease-activated receptor-1 (PAR-1) on extravascular cells by the coagulants, thrombin [8] and FXa [9], and the fibrinolytic mediator, plasmin [10] (Figure 1). The proteases which convert plasminogen into plasmin, urokinase- and tissue-type plasminogen activators (uPA and tPA, resp.), also directly signal through receptors, such as uPAR [11] and LDL receptor-related protein 1 (LRP-1) [12], involving integrin recruitment, to mediate proremodelling activities (Figure 1). Other extravascular actions of coagulants and fibrinolytic proteases are less direct, such as those of plasmin, which involve the formation of fibrin degradation products (FDPs) [13] or activation of matrix metalloproteinases (MMPs) and subsequent release of matrix-bound growth factors (transactivation) [14] (Figure 1).

## 2. Extravascular Coagulation in Disease 

Extravascular accumulation of fibrin, formed by the coagulation cascade, occurs in a number of diseases [2–7]. In tissue injury and inflammation, plasma containing FVII and FX leaks into the extravascular compartment [15–18]. FVII, combined with surface bound tissue factor (TF), which is formed by mesenchymal, epithelial, and inflammatory cells, transforms FX into the serine protease, FXa. The latter, combined with factor V (FV), activates thrombin, which in turn converts fibrinogen into fibrin. Abundant extravascular fibrin is a specific hallmark of lung injury and disease including acute lung injury (ALI) [2], asthma [3], and idiopathic pulmonary fibrosis (IPF) [4]. Increased levels of coagulant factors or activity are also detected in the induced sputum and bronchial lavage fluid of patients with respiratory disease [19, 20], particularly after exacerbation following rhinovirus infection [21]. Furthermore, endogenous FX is expressed in the lung tissue of IPF patients, localized to alveolar epithelial cells, macrophages, and myofibroblasts in fibrotic foci [22]. In rheumatoid arthritis, fibrin accumulates within inflamed hyperplastic synovial tissue and fluid of arthritic joints [6]. Deposits of insoluble fibrin on synovial membranes and pannus relate to the progression of arthritis [23]. In vascular disease, fibrin is present in normal arterial intima and in atherosclerotic lesions, particularly early proliferative, gelatinous-lesions [5]. In cancer, fibrin is detected surrounding carcinoma cells, particularly at the interface with surrounding stromal cells and blood vessels [7]. In disease, the deposition of fibrin into the extracellular matrix serves as a scaffold to support proliferation, migration, and growth of either mesenchymal (i.e., smooth muscle and fibroblasts) or tumor cells. In respiratory disease, excess accumulation of airspace fibrin is detrimental as it inactivates surfactant [24, 25]. In rheumatoid arthritis, fibrin becomes autoantigenic by the posttranslational modification, citrullination, possibly contributing to inflammation via a TLR-4 pathway [26].

## 3. Extravascular Fibrinolysis in Disease 

Fibrinolysis is the counterpart of coagulation. The key mediator of fibrinolysis is plasmin, which is formed by the proteolytic activation of plasminogen by either tPA or uPA [27, 28]. Plasmin function actually differs depending on where it is formed and by which activator [29]. Fibrinolysis* per se* is associated with tPA, which unlike uPA shows fibrin-enhanced proteolytic activity [25]. In interstitial tissue, uPA is the predominant means of plasminogen activation, contributing to pericellular proteolysis and cell activation [30]. Whilst plasmin has an important role in the resolution phase of wound-repair processes in damaged tissue by degrading fibrin [31] or by activating structural and inflammatory cells [14, 32–34], excessive formation of plasmin is potentially harmful. In vascular disease and injury, extravascular plasminogen activation is considered to contribute to tissue remodelling in the vascular wall by stimulating the proliferation and migration of vascular smooth muscle cells in neointima formation [35, 36]. In rheumatoid arthritis, synovial levels of fibrin D-dimer, a measure of fibrinolysis, correlates with disease severity and response to therapy [37, 38]. Acute tPA-mediated plasmin formation is a critical component of extravascular proteolytic damage in immature brains caused by hypoxia-ischemia [39]. In lung injury and disease, whilst suppressed tPA-mediated fibrinolysis contributes to the accumulation of airspace fibrin [40], increased uPA activity in the interstitium of damaged lung tissue favors temporal and localized increases in plasmin production [41–43]. The proteolytic activity of plasmin, whether via the formation of TLR-4-activating FDPs or by the activation of MMPs and/or PAR-1, contributes to inflammation and remodelling in disease.

## 4. Regulation of Coagulation and Fibrinolysis

Coagulation and fibrinolysis in physiological wound repair are highly regulated and integrated processes. The important negative regulator of coagulation, thrombomodulin, binds thrombin to prevent it from cleaving fibrinogen or activating PAR-1. Thrombomodulin-thrombin complexes also activate the anticoagulant, protein C. Hereditary deficiency of protein C is an established risk factor for venous thrombosis [44], as activated protein C (APC) cleaves and inactivates the coagulants, FVa and factor VIIIa (FVIIIa). Plasminogen and plasminogen activator coreceptors that accelerate and localize plasmin formation to the cell surface, such as uPAR and the annexin A2 heterotetramer (AIIt) [28], are important regulators of fibrinolysis. Fibrinolysis is negatively regulated by the serpin, plasminogen activator inhibitor 1 (PAI-1), which covalently binds to and inactivates plasminogen activators. PAI-1 levels are higher in many respiratory diseases [45–48]. Direct effects of PAI-1 on cells, independent of plasmin formation, may also contribute to disease pathology [49]. Another serpin, *α*2-antiplasmin, inactivates plasmin by binding its soluble but not fibrin-bound form [50]. Hyperfibrinolytic bleeding occurs in a number of diseases including chronic liver disease as a consequence of decreased production or loss of *α*2-antiplasmin [51].

There are a number of interaction points between coagulation and fibrinolysis. Thrombomodulin-bound thrombin activates thrombin activated fibrinolysis inhibitor (TAFI). This carboxypeptidase removes C-terminal lysine and arginine residues on fibrin, reducing plasminogen binding, hence activation and subsequent break down of fibrin. Increased levels of activated TAFI are associated with a number of diseased states including cardiovascular [52] and inflammatory bowel [53] diseases. TAFI can also cleave and inactivate uPA whereas plasmin can cleave and inactivate a number of coagulants including FVa, FVIIIa, and FXa. Plasmin proteolysis of FXa reveals a cryptic binding site for tPA in the cleaved product (denoted as *β*FXa) [54]. As binding of *β*FXa to tPA accelerates fibrinolysis [55], FXa-proteolysis is a pivotal switch from coagulation to fibrinolysis.

## 5. PAR Activation

**Figure 2:**
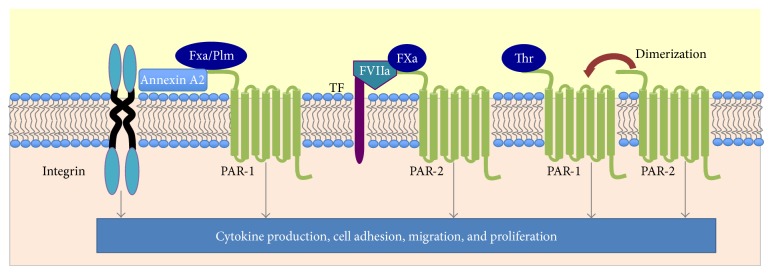
PAR signalling by coagulant and fibrinolytic proteases. Thrombin (thr), FXa, FVIIa, and plasmin (Plm) can signal via different combinations of PARs, coreceptors (e.g., *α*_9_ *β*_1_-integrin), and adaptors (e.g., annexin A2). Modulation of signalling may also involve PAR dimerization.

The PARs (PAR-1, -2, -3, and -4) are a G protein-coupled receptor family, activated by proteolysis of the N-terminus to reveal a tethered ligand. PAR-1 is the prototypical receptor of thrombin (and FXa) (Figure 2), although PAR-3 and -4 can be activated by thrombin also. PAR-2 is activated by complexes of either TF:FVIIa or TF:FVIIa:FXa [9, 56] (Figure 2). PARs are expressed in inflammatory cells including macrophages, mast cells, and eosinophils [57–60] and extravascular structural cells including epithelial, smooth muscle, and fibroblast cells [61]. Levels of PARs are increased in structural cells in fibrotic lung disease [62], and targeting PAR-1 reduces pulmonary inflammation and fibrosis in mouse models of lung injury and disease [22, 59, 63]. Furthermore, PAR-1 activation elicits increased cytokine and collagen expression [22, 64, 65] and proliferation [66] of lung mesenchymal cells. The upregulation of both PAR-1 and PAR-2 in vascular smooth muscle cells following injury and activation by hemostatic proteases is considered to contribute to the pathogenesis of atherosclerosis [67]. PAR-1, along with PAR-4, also appears to play an important role in cancer, mediating thrombin-evoked tumor cell migration [68]. In rheumatoid arthritis, PAR-1 and PAR-2 expression is increased in synovial fibroblasts, although it appears PAR-2 is the primary mediator of synovial fibroblast growth, invasion, and cytokine production [69].

## 6. PAR Coreceptors and Dimerization

PAR signalling is modulated by coreceptors and adaptors. An example is the dependency of the PAR-1-mediated cytoprotective effects of the anticoagulant, APC for the endothelial PC receptor (EPCR) or C11b integrin [70, 71]. Whilst plasmin has ~10 times less affinity for PAR-1 than thrombin [8], integrin coreceptors augment plasmin-evoked PAR-1 activation [10]. Binding to *α*_9_ *β*_1_ integrin localizes plasmin to the cell surface and protects it from *α*2-antiplasmin inhibition, increasing PAR-1 activation, whilst also activating pathways downstream of *α*_9_ *β*_1_ integrin through integrin-linked kinase (ILK) [10] (Figure 2). Annexin A2, a mediator of plasmin-stimulated cytokine production in macrophages and smooth muscle cells [14, 33, 72], also binds and protects plasmin from *α*2-antiplasmin inhibition [73]. Annexin A2 may facilitate plasmin localization to the cell surface and subsequent PAR-1 and integrin coactivation [74]. Annexin A2 also binds FXa to augment FXa-mediated activation of PAR-1 [9] (Figure 2).

## 7. PAR Dimerization

PAR-PAR interactions, such as PAR-2 transactivation by PAR-1, also modulate cellular responses evoked by coagulant and fibrinolytic proteases [75]. PAR-1 activation in tumor cells stimulates either rapid uPA release or increased plasminogen activator inhibitor-1 (PAI-1) expression, depending on whether PAR-1 is dimerized to PAR-2 [76]. Cooperative PAR-1 and PAR-4 signalling contributes to thrombin-mediated cancer cell migration [68]. Figure 2 summarizes the different configurations of PARs and coreceptors in mediating the actions of coagulants and plasmin.

## 8. Growth Factor Receptor Transactivation

Plasmin catalyzes the proteolytic activation of MMPs, which not only can activate PAR-1, but release the otherwise latent forms of growth factors such as epidermal growth factor (EGF) and TGF-*β* [77, 78]. Plasmin is involved in the activation of a number of MMPs including MMP-1, MMP-2, MMP-3, MMP-9, MMP-13, and MMP-14 [79]. Plasminogen activation by smooth muscle cells and fibroblasts is associated with MMP activation [80] and targeting the EGF-receptor (EGFR) or MMPs attenuates plasmin(ogen)-stimulated proliferation [14]. The effects of plasmin(ogen) on EGFR signalling are contributed by heparin-binding EGF, an EGFR ligand, which is released from cell surface heparan sulphate proteoglycan by MMP-mediated proteolysis. In a manner similar to EGFR transactivation, plasmin-stimulated mobilization of matrix-bound TGF-*β* contributes to collagen synthesis in smooth muscle cells in a manner involving TGF-*β* receptor signalling [77]. Additionally, the plasmin-activated MMP-1 and MMP-13 also cleave the N-terminal exodomain of PAR-1, but at sites alternative to those of thrombin and FXa, to elicit distinct cellular responses thought to be relevant in cancer and rheumatoid arthritis pathology [81].

## 9. Plasmin-Independent Actions of uPA

Increased levels of uPA occur in many pathologies, including chronic respiratory and vascular disease [45, 46, 48, 82, 83], rheumatoid arthritis [84], and cancer [85]. Aside from its role in plasmin formation, uPA also elicits cellular responses via binding its receptor, uPAR, which lacks a transmembrane or intracellular domain. The aminoterminal fragment of uPA interacts with uPAR to activate coreceptors including the formyl-peptide receptor 2 (FPR2) [86], EGFR [87], and integrins [11] to regulate migration, chemotaxis, and cytokine production. Integrin binding extracellular matrix (ECM) proteins such as fibulin 5 and vitronectin modulate uPA-uPAR signalling [88]. In an uPAR-independent manner, the kringle domain of uPA interacts with the *α*_v_ *β*_1_-integrin to elicit intracellular signalling and cell migration [89].

## 10. Fibrin(ogen) Fragments in Inflammation 

The coagulation and plasminogen activation systems also contribute to inflammation by their respective roles in the formation and subsequent break down of fibrin. Fibrinogen cleavage by thrombin releases the fibrinopeptides A and B, which are potent chemoattractants for neutrophils, monocytes, and macrophages [90, 91]. Fibrin and FDPs activate inflammatory and/or structural cells via binding TLR-4 [13] or CD11b/CD18 integrins [92] and regulate smooth muscle cell migration via binding *α*_5_ *β*_3_ integrin [93]. Fibrin D-dimers, a biomarker for hyperfibrinolytic disorders such as disseminated intravascular coagulation (DIC), stimulate increased cytokine production in peripheral blood monocytes and leukemia cell lines [94, 95]. Fibrin fragment E induces leukocyte cytokine expression and migration by binding vascular endothelial-cadherin and monocyte and neutrophil migration by binding CD11c [96]. The small FDP, B*β*15–42, is a potent chemoattractant for neutrophils and fibroblasts and induces cytokine expression in human oral squamous cell carcinoma cells [97]. However, B*β*15–42 also has anti-inflammatory and immunosuppressive actions which are considered to be protective in ischemia reperfusion injury and hemorrhagic shock [98, 99]. FXa may also be an endogenous activator of the innate immune system as FXa is required for adenoviral activation of TLR-4/MyD88 signalling in host cells [100]. TLR-4 activation by FXa may involve its binding partner, annexin A2 [9]. Annexin A2 activates TLR-4 to regulate smooth muscle cell proliferation and macrophage cytokine production [14, 101] and is an “adaptor” for TLR-4 in a multiprotein signalling scaffold on endothelial cells in antiphospholipid syndrome [102]. Interestingly, plasmin also has a role in the innate immune system by inducing the dissociation of annexin A2 from the extracellular heterotetrameric complex it forms with S100A10, allowing monomeric annexin A2 to activate TLR-4 [103].

## 11. Inflammatory Actions of Coagulant Proteases Are Glucocorticoid-Insensitive 

Synthetic glucocorticoids (GCs) are the most effective anti-inflammatory therapy for disease including rheumatic diseases, allergy, asthma, and sepsis. However, GC resistance limits the therapeutic response of GCs in certain chronic inflammatory diseases including severe asthma, IPF, and cancer [104]. GC resistance has been attributed to cellular microenvironment changes, that is, alterations in the ECM, as a consequence of chronic inflammation [105]. Thrombin is a GC-insensitive mediator of inflammation and remodelling based on its response requiring 100-fold greater concentrations of dexamethasone than those of IL-1*α* and other cytokines [106]. Integrins may render the PAR-mediated actions of coagulant proteases insensitive to GCs. *β*_1_-integrin mediates diminished GC responsiveness in smooth muscle cells [107], and the *β*_6_-integrin is responsible for impaired skin wound healing caused by GCs [108]. Interestingly, the integrin binding annexin A2 has a role in GC resistance in leukemia [109].

## 12. Selectively Targeting Coagulation Proteases as Therapy

Targeting coagulation protease activity or signalling is a potential treatment for inflammatory disease. However, anticoagulant therapies, including selective small molecule FXa inhibitors used to treat thrombotic diseases (e.g.,* Apixaban*), are typically associated with potentially fatal bleeding risks [110]. Furthermore, orally administered PAR-1 inhibitors such as* Vorapaxar*, whilst not affecting fibrin formation, suppress thrombin-stimulated platelet aggregation, hence interfering with hemostasis.* Vorapaxar* is used for prevention of secondary thrombotic cardiovascular events in patients with a prior myocardial infarction [111], despite having been withdrawn from phase III trials as treatment for acute coronary syndrome due to bleeding complications [112]. Improved inhibitors, that selectively target the extravascular cell-mediated actions of coagulant proteases without disrupting hemostasis, are likely to have greater therapeutic windows as treatment for inflammation. The design of such inhibitors may take advantage of coreceptors and adaptors that differentiate PAR responses in platelet and endothelial cells as compared to inflammatory and extravascular structural cells.

## 13. Therapeutic Potential of Targeting Fibrinolytic Proteases

Selective targeting interstitial plasmin formation is another potential strategy to treat chronic inflammatory disease. Highly selective small molecule inhibitors of uPA such as* WX-UK1* have been evaluated in preclinical and clinical studies to assess whether uPA is a potential target to treat cancer [113–115]. In clinical trial programs, oral administration of uPA inhibitors was assessed to be well-tolerated and provided promising results, which greatly increase their translational potential as anti-inflammatory therapies. Functional blocking antibodies for uPA and/or uPAR have also been evaluated in preclinical studies of cancer, hepatic fibrinolysis, and ALI [116–118], providing evidence for the feasibility of targeting uPA using antibody-based strategies. Annexin A2, a regulator of plasmin formation and signalling, is implicated in cancer with its levels elevated in various tumors [119, 120]. The systemic administration of annexin A2 antibody inhibits tumour growth and metastasis in murine cancer models* in vivo* without detectable toxicity [121, 122].

## 14. Conclusion

Coagulant and fibrinolytic proteases evoke proinflammatory and remodelling actions in disease. Coagulants, plasminogen activators, plasmin, and plasmin-activated MMPs evoke cell-mediated responses via receptors (e.g., PARs and uPAR) and coreceptors (e.g., integrins and FPR2). Plasmin also indirectly contributes to inflammatory processes by forming FDPs or by growth factor receptor transactivation. Targeting the inflammatory actions of coagulant and/or fibrinolytic proteases without disrupting hemostasis is a strategy that may be beneficial in the treatment of inflammatory disease.

## Abbreviations

ALI: Acute lung injury
EGF: Epidermal growth factor
ECM: Extracellular matrix
FV: Factor V
FVII: Factor VII
FX: Factor X
FXa: Factor X activated
FDPs: Fibrin degradation products
FPR2: Formyl-peptide receptor 2
GCs: Glucocorticoids
IPF: Idiopathic pulmonary fibrosis
LRP-1: LDL receptor-related protein 1
MMP: Matrix metalloproteinases
PAR: Protease-activated receptor
TF: Tissue factor
tPA: Tissue type-plasminogen activator
TLR-4: Toll-like receptor-4
TGF-*β*: Transforming growth factor-*β*
uPA: Urokinase plasminogen activator
uPAR: uPA receptor.
